# Influence of Bone Marrow-Derived Mesenchymal Stem Cell Therapy on Oxidative Stress Intensity in Minimally Conscious State Patients

**DOI:** 10.3390/jcm9030683

**Published:** 2020-03-03

**Authors:** Katarzyna Jezierska-Wozniak, Emilia Sinderewicz, Wioleta Czelejewska, Pawel Wojtacha, Monika Barczewska, Wojciech Maksymowicz

**Affiliations:** 1 Department of Neurosurgery, Laboratory of Regenerative Medicine, School of Medicine, Collegium Medicum, University of Warmia and Mazury in Olsztyn, Warszawska 30 Str., 10-082 Olsztyn, Poland; emilia.sinderewicz@uwm.edu.pl (E.S.); wioleta.czelejewska@uwm.edu.pl (W.C.); 2 Department of Neurosurgery, School of Medicine, Collegium Medicum, University of Warmia and Mazury in Olsztyn, Warszawska 30 Str., 10-082 Olsztyn, Poland; monika.barczewska@uwm.edu.pl (M.B.); maksymowicz@interia.pl (W.M.); 3 Department of Industrial and Food Microbiology, Faculty of Food Sciences, University of Warmia and Mazury in Olsztyn, Plac Cieszynski 1 Str., 10-726 Olsztyn, Poland; pawel.wojtacha@uwm.edu.pl

**Keywords:** minimally conscious state, mesenchymal stem cells, oxidative stress, traumatic brain injury

## Abstract

Neurological disorders, including minimally conscious state (MCS), may be associated with the presence of high concentrations of reactive oxygen species within the central nervous system. Regarding the documented role of mesenchymal stem cells (MSCs) in oxidative stress neutralization, the aim of this study is to evaluate the effect of bone marrow-derived MSC (BM-MSC) transplantation on selected markers of oxidative stress in MCS patients. Antioxidant capacity was measured in cerebrospinal fluid (CSF) and plasma collected from nine patients aged between 19 and 45 years, remaining in MCS for 3 to 14 months. Total antioxidant capacity, ascorbic acid and ascorbate concentrations, superoxide dismutase, catalase, and peroxidase activity were analyzed and the presence of tested antioxidants in the CSF and plasma was confirmed. Higher ascorbic acid (AA) content and catalase (CAT) activity were noted in CSF relative to plasma, whereas superoxide dismutase (SOD) activity and total antioxidant capacity were higher in plasma relative to CSF. Total antioxidant capacity measured in CSF was greater after BM-MSC transplantations. The content of ascorbates was lower and CAT activity was higher both in CSF and plasma after the administration of BM-MSC. The above results suggest that MSCs modulate oxidative stress intensity in MCS patients, mainly via ascorbates and CAT activity.

## 1. Introduction

Traumatic brain injury (TBI), structural brain lesions, acute endocrine-metabolic disorders, neuronal dysfunctions, and psychogenic unresponsiveness, among many others, provoke consciousness disturbances [1,2,3] which, depending on the severity and duration, may be distinguished in coma and states following coma: minimally conscious state (MCS) and vegetative state (VS) [4]. According to the definition by the Aspen Neurobehavioral Conference Workgroup, MCS is a condition of severely altered consciousness in which minimal, but definite, behavioral evidence of self or environmental awareness is demonstrated on a reproducible or sustained basis by one or more of behaviors such as following simple commands, gestural or verbal yes/no responses, or purposeful behavior [4].

Consciousness disturbances are accompanied by an elevated level of oxidative stress, manifesting a high concentration of reactive oxygen species (ROS). Excessive ROS production and losing the scavenging capacity of the antioxidant response system, lead to extensive protein oxidation and lipid peroxidation, which seem to be particularly dangerous in a lipid-rich content nervous system [5,6,7]. These changes provoke oxidative damage, cellular degeneration, and functional decline of the central nervous system (CNS) [8,9]. It has been demonstrated that high ROS concentrations reportedly diminish long-term potentiation and synaptic signaling as well as brain plasticity mechanisms [10,11]. This condition is considered to be a state of oxidative stress and poses a real threat to the normal functioning of the brain [12,13,14]. 

The natural defense mechanisms against oxidative stress described in the nervous system can be divided into two groups: low molecular weight antioxidants action and the antioxidant enzyme system [15,16]. The first mechanism includes glutathione, uric acid, and vitamins (e.g., ascorbic acid (AA)). Due to its high concentration in neurons and documented function as a neuromodulator and ROS scavenger, AA is speculated as a general antioxidant in the central nervous system [17]. Its role is to counteract oxidants by the chelation of transition metals [18]. Among the antioxidant enzymes present in CNS, glyoxalase, glutathione reductase, glutathione peroxidase (GPx) and superoxide dismutase (SOD), and catalase (CAT) families have been described [19]. Members of the SOD family, including Mn-SOD and Cu-Zn-SOD, enable the dismutation of superoxide radicals to hydrogen peroxide (H_2_O_2_), which is removed by other enzymes: CAT and GPx [20].

Although the prevalence of MCS has been estimated to be 112,000 to 280,000 adult and pediatric cases [4,21] and a number of clinical trials have been performed, there is currently no effective therapy for MCS [22,23,24]. One promising innovative therapeutic approach seems to be mesenchymal stem cell therapy. The potential of mesenchymal stem cells (MSCs) to differentiate into mature cells of different lineages, and above all, the possibility of expanding in vivo and immunoregulatory properties [25], makes them an important tool in cell therapy, regenerative medicine, and neurodegenerative disorders. The latest study documented that MSC transplantation suppressed oxidative stress in Alzheimer’s disease [26]. Studies on cell cultures and animal models show that MSC transplantation improved motor and receptive functions after cerebral stroke [27,28,29], TBI, spinal cord injury [30,31], and promoted nerve remodeling [32]. In rodents, injection of MSC after TBI leads to neuroprotection by maintaining blood–brain barrier integrity, promoting activated microglial apoptosis, and improving cognitive function [33,34,35]. In ischemic rodents, MSC transplantation enhanced functional recovery, decreased the volume of brain infarction, and promoted the expression of neurotrophic factors [36,37]. Similarly, in humans with ischemic stroke, transplantation of MSC resulted in reduced scores on the Health Stroke Scale, indicating a transition from moderate to minor stroke [29]. It was also proven that MSCs are resistant to conditions generating oxidative stress, such as ionizing radiation [38]. Moreover, MSCs were not susceptible to cell death induced by oxidative stress. It was documented that MSCs exposed to oxidative stress revealed a low concentration of intracellular reactive species together with a high expression of enzymes required to manage the oxidative stress, such as CAT and GPX [39].

Considering that recent investigations documented the ability of MSCs in oxidative stress neutralization and the wide range of MSC applications in regenerative medicine, it is hypothesized that autologous transplantation of bone marrow-derived MSCs (BM-MSCs) decreases the range of oxidative stress in patients in MCS. As far as it is known, this is the first study investigating the influence of BM-MSC therapy on oxidative stress in MCS patients.

## 2. Material and Methods

The study was conducted by the Department of Neurosurgery and Laboratory of Regenerative Medicine Stem Cells Bank, School of Medicine, Collegium Medicum, University of Warmia and Mazury in Olsztyn, Poland and the University Clinical Hospital in Olsztyn, Poland. The study was approved by the Bioethical Committee of the School of Medicine, University of Warmia and Mazury in Olsztyn, Poland (ethical approval No. 19/2017 in April 2017). This study was performed in accordance with the Declaration of Helsinki. 

### 2.1. Patient Characteristic

Nine patients aged between 19 and 45 years remaining in MCS for 3 to 14 months qualified for BM-MSC therapy. The characteristics of these patients are presented in Table 1. For the purposes of the experiment, a diagnostic protocol based on magnetic resonance imaging, functional magnetic resonance imaging, electroencephalography, and analysis of the 99mTc-hexamethylpropylene amine oxime (HMPAO) single photon emission tomography/computed tomography (SPECT-CT) cerebral perfusion factor to the differentiation of consciousness disorders was used.

### 2.2. Bone Marrow Collection and Isolation of MSC

Approximately 200 mL of bone marrow were obtained from each patient (*n* = 9) in local anesthesia from the posterior iliac crest.

The culture of purified BM-MSCs was prepared under aseptic conditions according to Good Manufacturing Practice (GMP) procedures (European Medicines Agency, 1999). Briefly, a phosphate-buffered saline (PBS)-diluted (Gibco, cat no. 18912-014, Thermo Fisher Scientific, Waltham, MA, USA) cell fraction of bone marrow was layered over a Ficoll density gradient (1.077 g/mL, cat no. 17-1440-03, GE Healthcare, Boston, MA, USA), followed by centrifugation at 400 G at room temperature for 40 min. Nucleated cells were collected, diluted with two volumes of PBS, centrifuged twice at 200 G for 10 min, and finally resuspended in culture medium (Gibco DMEM/F-12, GlutaMAX^TM^, cat no. 10565018, Thermo Fisher Scientific, Waltham, MA, USA). Cells were plated and expanded in a T-150 flask (Falcon Blue Plug Seal Cap, cat no. 355000, Corning Inc, Corning, NY, USA) and grown at 37 °C and 5% CO_2_ [40]. After five days, the medium was replaced, and unattached cells were removed. After reaching 75% of confluence, cells were washed with PBS, harvested by trypsinization, and frozen in culture medium containing 10% of dimethyl sulfoxide (DMSO, cat no. D2650, Sigma-Aldrich, St. Louis, MO, USA).

The cell surface marker expression was determined for all patients to certify the safety, identity, potency, and the pharmaceutical grade of the MSCs, as well to satisfy the GMP regulatory process criteria. The set of primary antibodies (The BD Stemflow™ Human MSC Analysis Kit, cat. no. 562245, BD Biosciences, Franklin Lakes, NJ, USA) was used to determine the MSC phenotype, according to MSC features established by the International Society for Cellular Therapy guidelines [41]. Flow cytometry was performed using a fluorescence activated cell sorter (BD Facs Aria II, BD Biosciences, Franklin Lakes, NJ, USA) and the results were analyzed with DIVA software.

Before transplantation, the cells were thawed in a basal culture medium without serum, detached, and washed three times with PBS 1× containing 1% human albumin (Alburex 5, CSL Behring GmbH, Marburg, Germany). The number of cells was determined by analysis in a Bürker chamber (Heinz Herenz Medizinalbedarf GmbH, Hamburg, Germany) with Trypan blue (cat. no T8154, Sigma-Aldrich, St. Louis, MO, USA) staining. A mean of 20 × 10^6^ cells were injected intrathecally during the neurosurgical procedure by the neurosurgeon. The MSCs were administered to the patients three times every two months.

The culture of purified MSCs was prepared under aseptic Good Manufacturing Practice conditions by the European Medicines Agency in 1999, where manufacturing facilities maintain a clean and hygienic manufacturing area in controlled environmental conditions. All manufacturing processes were clearly defined, controlled, and validated to ensure consistency and compliance with specifications. The laboratory has all the approvals and certificates required by Polish and European law.

### 2.3. Plasma and Cerebrospinal Fluid Collection

Immediately before MSC transplantation, 8 mL of cerebrospinal fluid (CSF) and up to 2 h before MSC transplantation, 8 mL of plasma sample were collected. CSF was collected from patients by lumbar puncture made by a neurosurgeon. Plasma and CSF collected before the first administration of MSCs provided control values for the analyzed parameters. The collected CSF and plasma samples were aliquoted and frozen at −80 °C for further analysis.

### 2.4. Antioxidant Capacity Measurement

Antioxidant capacity was measured in CSF and plasma collected from nine patients by examination of total antioxidant capacity (Antioxidant Assay Kit, cat. no. CS0790, Sigma Aldrich, St. Louis, MO, USA), ascorbic acid and ascorbate concentrations (Ascorbic Acid Assay Kit, cat. no. MAK075, Sigma Aldrich, St. Louis, MO, USA), superoxide dismutase, and catalase activity (SOD Determination Kit, cat. no. 19160, Sigma Aldrich, St. Louis, MO, USA; Catalase Assay Kit, cat. no. CAT100, Sigma Aldrich, St. Louis, MO, USA, respectively), according to the manufacturer’s instructions. Peroxidase activity was measured by Peroxidase Activity Assay Kit (cat. no. MAK092, Sigma Aldrich, St. Louis, MO, USA) and methods described by Shannon et al. [42] and Maehly and Chance [43]. All measurements were performed in duplicate.

### 2.5. Statistical Analysis

**Figure 1:**
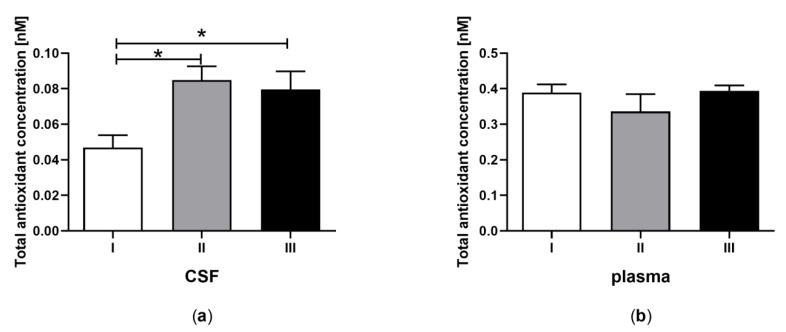
Total antioxidant capacity in cerebrospinal fluid (CSF) (Figure 1a) and plasma (Figure 1b) of the patients in MCS before (white bars) and after first (grey bars) and second (black bars) transplantation of bone marrow-derived (BM)-MSC. The statistical analysis was determined by a one-way ANOVA followed by Tukey’s multiple comparison test (*p < 0.05*). Asterisks indicate differences in the antioxidant capacity between examining groups (* *p <* 0.05).

**Figure 2:**
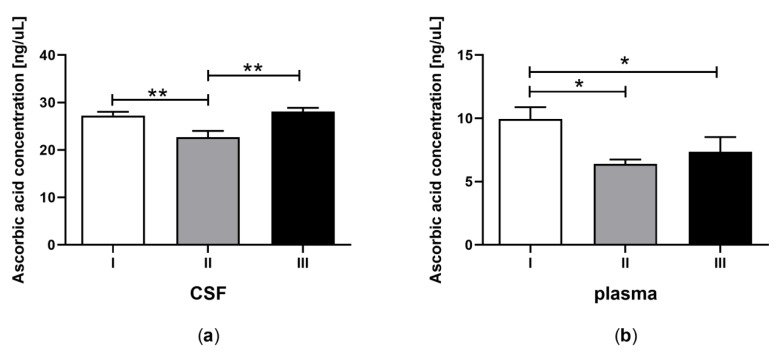
Ascorbic acid concentration in CSF (a) and plasma (b) of the patients in MCS before (white bars) and after first (grey bars) and second (black bars) transplantation of BM-MSC. The statistical analysis was determined by a one-way ANOVA followed by Tukey’s multiple comparison test (*p <* 0.05). Asterisks indicate differences in the antioxidant capacity between examining groups (* *p <* 0.05; ** *p <* 0.001).

**Figure 3:**
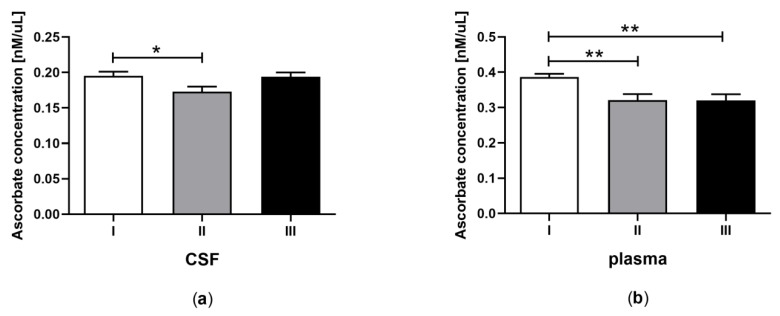
Total ascorbate concentration in CSF (a) and plasma (b) of the patients in MCS before (white bars) and after first (grey bars) and second (black bars) transplantation of BM-MSC. The statistical analysis was determined by a one-way ANOVA followed by Tukey’s multiple comparison test (*p* < 0.05). Asterisks indicate differences in the antioxidant capacity between examining groups (* *p <* 0.05; ** *p <* 0.001).

**Figure 4:**
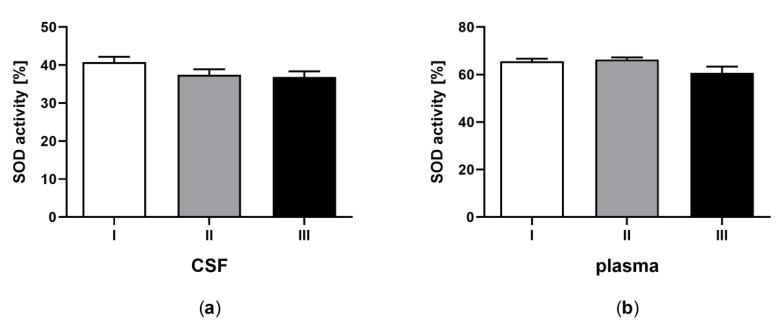
Superoxide dismutase (SOD) activity in CSF (a) and plasma (b) of the patients in MCS before (white bars) and after first (grey bars) and second (black bars) transplantation of BM-MSC. The statistical analysis was determined by a one-way ANOVA followed by Tukey’s multiple comparison test (*p* < 0.05).

**Figure 5:**
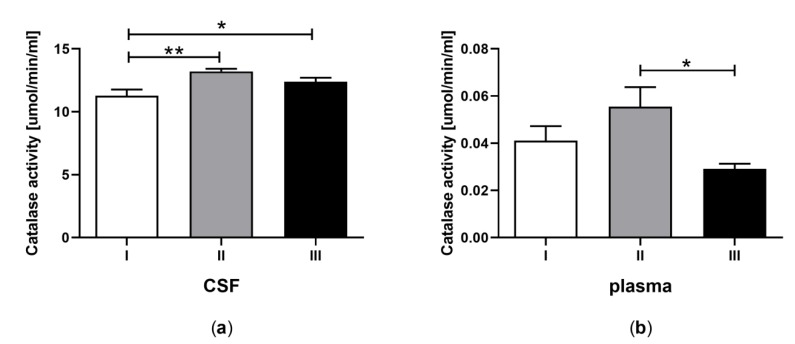
Catalase activity in CSF (a) and plasma (b) of the patients in MCS before (white bars) and after first (grey bars) and second (black bars) transplantation of BM-MSC. The statistical analysis was determined by a one-way ANOVA followed by Tukey’s multiple comparison test (*p <* 0.05). Asterisks indicate differences in the antioxidant capacity between examining groups (* *p <* 0.05; ** *p <* 0.001).

Statistical analyses were performed using GraphPad PRISM v. 8.0 software (GraphPad Software, Inc.). All experimental data are shown as the mean ± SEM, and the differences were considered significantly different at a 95% confidence level (*p* < 0.05). The analyses were performed using one-way ANOVA followed by Tukey’s multiple comparison test (Figure 1, Figure 2, Figure 3, Figure 4 and Figure 5) or Student’s *t*-test (Table 2).

## 3. Results

### 3.1. Total Antioxidant Capacity in CSF and Plasma of the Patients in MCS Undergoing BM-MSC Treatment

Total antioxidant capacity, determined by evaluation of the ABTS (2,2′-azino-bis(3-ethylbenzothiazoline-6-sulfonate) oxidation ability by active antioxidants in CSF (Figure 1a) and plasma (Figure 1b) was examined for patients in MCS undergoing BM-MSC therapy. The antioxidant concentration in CSF was higher after the first and second application of BM-MSC than before treatment (Figure 1a, *p < 0.05*). In plasma, statistical differences in antioxidant capacity between examined groups were not found (Figure 1b, *p > 0.05*). Antioxidant capacity was four-fold greater in plasma compared to CSF (Table 2A, *p < 0.0001*).

### 3.2. Ascorbic Acid Concentration in CSF and Plasma of the Patients in MCS Undergoing BM-MSC Treatment

The concentration of AA in CSF (Figure 2a) and plasma (Figure 2b) was determined for patients in MCS undergoing BM-MSC therapy. A lower concentration of AA was detected in CSF after the first application of BM-MSC (Figure 2a, *p* < 0.05). The AA content was equalized to the control value after the second application of MSC (Figure 2a, *p* > 0.05). In the plasma, the AA concentration was greater before MSC treatment compared to the content after the application of BM-MSC (Figure 2b, *p* < 0.05). The AA concentration was three-fold greater in CSF towards plasma (Table 2B, *p* < 0.0001).

### 3.3. Total Ascorbate Concentration in CSF and Plasma of the Patients in MCS Undergoing BM-MSC Treatment

The concentration of ascorbates in CSF (Figure 3a) and plasma (Figure 3b) was examined for patients in MCS undergoing BM-MSC therapy. The total concentration of ascorbates in CSF was lower after the first application of BM-MSC than before treatment (Figure 3a, *p* < 0.05). In plasma, the total concentration of ascorbates was lower after the first and second application of BM-MSC than before therapy (Figure 3b, *p* < 0.05). The total content of ascorbates was two-fold greater in plasma compared to CSF (Table 2C, *p* < 0.0001).

### 3.4. Superoxide Dismutase Activity in CSF and Plasma of the Patients in MCS Undergoing BM-MSC Treatment

The activity of SOD in CSF (Figure 4a) and plasma (Figure 4b) was determined for patients in minimal MCS undergoing BM-MSC therapy. The SOD activity was not statistically different between the control and applications of BM-MSC, both in CSF (Figure 4a, *p >* 0.05) and plasma (Figure 4b, *p >* 0.05). The SOD activity was greater in plasma in comparison to CSF (Table 2D, *p* < 0.0001).

### 3.5. Catalase Activity in CSF and Plasma of the Patients in MCS Undergoing BM-MSC Treatment

The CAT activity in CSF (Figure 5a) and plasma (Figure 5b) was determined for patients in MCS undergoing BM-MSC therapy. The CAT activity measured in CSF was greater after the first and second application of BM-MSC than before treatment (Figure 5a, *p* < 0.05), whereas in plasma it was lower after the second application of BM-MSC in comparison to the first application (Figure 5b, *p <* 0.05). The CAT activity was 200-fold greater in CSF in comparison to plasma (Table 1E, *p <* 0.0001).

### 3.6. Peroxidase Activity in CSF and Plasma of the Patients in MCS Undergoing BM-MSC Treatment

The activity of peroxidase in CSF and plasma was measured for patients in MCS undergoing BM-MSC therapy. The results gained by using different protocols did not confirm the presence of active peroxidases in the examined material.

## 4. Discussion

Oxidative stress accompanies neurodegenerative disorders, causing neuronal dysfunctions and vascular damage [30,44]. The latest studies have shown that the level of oxidative stress is elevated in disorders of consciousness [7]. The intensity of oxidative stress can be determined in three ways: (i) by evaluation of ROS level; (ii) by direct evaluation of the concentration of antioxidants (enzymatic and non-enzymatic); and (iii) by measuring oxidative stress biomarkers, defined as a biological molecule, whose chemical existence is modified by ROS [45]. As the definition of oxidative stress is a lack of the proper quantity of tools dismissing ROS [6,46,47,48] and according to research, showing the ability of MSCs to reduce oxidative stress intensity and increase content or activity of enzymes neutralizing ROS [39], it could be supposed that MSC therapy may contribute to greater antioxidant ability of patients in MCS. We consider three ways of MSC activity: (i) activation of antioxidant mechanisms in CNS cells, inoperative in MCS; (ii) intensification of antioxidants transported from plasma to CSF; and (iii) activation of immunomodulatory role of examining antioxidants. As the concentration of antioxidants in plasma did not change significantly after BM-MSC application and because of the constant, greater concentration or activity of examining molecules in plasma in comparison to CSF, the activation of temporary inactive mechanisms in CNS seems to be the most possible.

To select the potential mechanism of BM-MSC action involved in oxidative stress reduction in MCS, the concentration or activity of molecules regulating this process in CNS was examined. Among them was ascorbic acid (AA), which participates in neuromodulation [49,50], extension of synaptic reactivity [51], catecholamine biosynthesis, and modulation of cell proliferation and differentiation [17,52]. Ascorbic acid (as a source of electrons having the ability to neutralize free radicals formed in the CNS [53]) is also involved in antioxidant protection [54,55,56]. The AA content and its antioxidative role were extensively investigated in numerous neurodegenerative disorders, including meningitis [57], Alzheimer’s disease [58,59], neurocysticercosis [60], Creutzfeldt–Jakob disease [61], aseptic encephalopathy [62], amyotrophic lateral sclerosis [63], and Parkinson’s disease [64]. However, to the best of our knowledge, this is the first study evaluating the relationship between the concentration of AA and BM-MSC therapy of MCS. 

The analysis of AA concentrations in CSF from controls and patients with neurological disorders did not show significant differences [63,65], which is consistent with the current results. This study found that the concentration of AA in CSF of patients in MCS was 23 ± 10 ng/ul, which is approximately equal to 133 ± 58.8 µmol/L, measured in CSF of healthy humans [66]. On the other hand, in CSF of patients with septic encephalopathy, decreased levels of ascorbates were observed [62] and these results correlated with neurologic symptoms. Similarly, a reduced concentration of total AA in the CSF was found in patients with head trauma, increased intracranial pressure, cerebral tumors [67], intracranial hemorrhage, or head trauma [68], indicating extensive consumption of AA in severe oxidative stress. Moreover, the AA level was inversely correlated with the diameter of the lesion and the number of neurological disabilities evaluated by the Glasgow Coma Scale (GCS) [68]. In turn, de Menezes et al. [57] showed increased AA content in CSF of patients with aseptic and bacterial meningitis. The above studies suggest that the role of AA in the CNS depends on the type of disorder. As MCS is mostly a long-term state with varied etiology, it seems possible that, along with the duration of disease, the AA content in CSF equalizes to the initial level, and the concentration of AA in the CNS is relatively stable in comparison with other organs [69]. The presence of an adaptive mechanism to oxidative stress was previously suggested in patients with head injury. It was documented that the level of lipid peroxidation was reduced over time and was correlated with an improvement in GCS scores [70]. Moreover, a high level of AA was present in neurons despite a concentration gradient promoting diffusion from the brain to peripheral tissues [58] and correlated with density of neurons, which probably store AA in the brain [69]. 

The current study found that AA concentration in CSF was significantly lower after the first autologous transplantation of BM-MSCs in comparison with the concentration before cell administration, which indicates that AA was intensively consumed in the CNS tissues in response to a BM-MSC application, and the level of oxidative stress accompanying MCS may be decreased in response to BM-MSC transplantation. Surprisingly, the effect of BM-MSC administration on AA concentration in CSF was compensated for after the second transplantation, which may be a result of a mechanism maintaining a high and relatively constant level of AA in the brain [17,58]. Moreover, the AA content decreased in plasma after BM-MSC administration, suggesting transport of the AA from plasma to peripheral tissues. The suspected effect of AA transport from plasma to the CNS as a response to oxidative stress is increased AA levels in CSF. In this study, the opposite result was observed, which may be a consequence of the bedridden patients’ diets, poor in natural sources of exogenous AA. Moreover, besides AA there may be another mechanism involved in oxidative stress regulation. Alho et al. [71] also suggested the presence of unidentified antioxidants in CSF and plasma, influencing the process of neurological diseases. Similar observations were obtained by a group examining another experimental therapy of consciousness disorders (electrical cervical spinal cord stimulation) resulting in lower free radical levels [72]. However, that the consumption of AA resulted from the elevated oxidative stress caused by mechanistic interference in the body during BM-MSC application cannot be excluded.

Although the concentration of AA in CSF is relatively constant in patients with neurodegenerative disorders [63,65], the CSF:plasma concentration ratio of AA may be a valuable tool to evaluate the role of AA role examining disorders or therapy. Bowman et al. [58] showed that despite its concentration in CSF and plasma, AA did not indicate Alzheimer’s disease progression; rather, the CSF:plasma AA ratio was correlated with the rate of cognitive decline, suggesting that this ratio is an indicator of AA availability in the brain. The current results documented that, apart from the higher AA content in plasma in the control in comparison to the concentration measured after both applications of BM-MSCs, the ratio of AA concentration in CSF to the AA concentration in plasma was changed from 3:1 to 4:1 after the first and second transplantations, which may indicate that AA sources were completed by the transport of AA from plasma to CSF. The concentration of AA is the most constant in the brain compared to the other organs [17,73,74,75] and because of that the CSF:plasma ratio may reflect the uptake, and thereby the activity of blood–CSF transporter [63]. As AA is not produced in human tissues, its efficient transport plays a pivotal role in providing appropriate levels in the CNS. It was documented that ascorbic acid is transported into the brain via CSF and epithelial cells of choroid plexus (appointed with sodium AA transporters) incorporating reduced forms of AA into the cytoplasmic membrane [75,76,77] and glucose transporters, uptaking its oxidized form as dehydroascorbic acid (DHA) [78,79]. This two-fold transport mechanism may explain the alteration of the ratio of CSF:plasma AA content. Ascorbic acid is mainly incorporated in neurons in its oxidized form [80], which modifies neuron functions [81,82]. According to the above data, the administration of BM-MSCs may indirectly alter neurons’ ability to regulate oxidative stress in MCS. Nevertheless, to confirm and clarify this hypothesis, further investigation is needed.

The current study also comprises an analysis of the total reduction ability in CSF and plasma, involving measurements of the concentrations of AA with other molecules as electron donors neutralizing ROS. Although similar trends in total ascorbate content as in the case of AA concentration were observed, the influence of BM-MSC application on anti-oxidation capacity was weaker in CSF and stronger in plasma of patients in MCS. Furthermore, the current study found a three times greater content of AA and two times lower concentration of all electron donors in CSF in comparison to plasma, which indicates the importance of AA in CNS homeostasis. Moreover, among electron donors there are also anions, being transitional forms of AA, leading to DHA formation, which is a transport type of AA [78,79]. Increased lipid peroxidation and reduced antioxidant activities in plasma were observed in Alzheimer’s disease [83,84], Creutzfeldt–Jakob disease [61], and acute ischemic stroke [85], proving oxidative mechanisms involved in the pathogenesis of these disorders. A positive correlation between oxidants, such as glutathione reductase level, and GCS scores was also found. The above results suggest that low weight molecules play an important anti-oxidative role in both CSF and plasma, however, AA is pivotal in oxidative stress regulation in the CNS, which is consistent with previous studies [54,55,56]. However, the great concentration of electron donors in plasma, caused by the response of the patient and consisting of the preparation of increased doses of AA for transport to CSF, cannot be excluded.

The appropriate level of ROS in the CNS is also maintained by the enzyme system, including SOD, CAT, and GPx. The only known function of SOD is the dismutation of O_2_^•-^ to H_2_O_2_. Catalase and GPx catalyze the conversion of H_2_O_2_ into oxygen and water. It is well known that three isoforms of SOD participate in the formation of the primary defense against ROS-mediated damage [86,87,88]. In this study, no changes in SOD activity were observed in either the plasma or CSF. There was lower SOD activity in CSF than in plasma, measured both before and after BM-MSC transplantation, which may indicate that the function of the blood–brain barrier in the analyzed group of patients has not been affected by the oxidative stress causing vascular leakage. Moreover, it was proven that the concentration of the main isoform of SOD—CuZn-SOD—in CSF is positively dependent on its concentration in the cytosol of neurons and the rates of basal background leakage and increased leakage from impaired cells, and conversely dependent on the rate of turnover of CSF [87]. In acute diseases such as stroke, when a large number of neurons are damaged, multiple-fold increases in the amount of CuZn-SOD in the CSF have been found [89,90]. Although MSC therapies are very promising in a number of neurodegenerative disorders [31,91,92,93], the current results suggest that BM-MSC therapy for MCS patients does not influence SOD activity.

The analysis of oxidative stress in neurodegenerative disorders has shown a short-term effect of enzymatic antioxidant defense. Higher CAT activity and lower total SOD activity was detected in the plasma of patients within the first 24–72 h of acute ischemic stroke onset compared to healthy controls and returned to control values within five days [94,95]. Likewise, it has been shown that on days 1–7 after aneurysmal subarachnoid hemorrhage CSF SOD levels were lower and serum malondialdehyde levels were higher in patients than in healthy controls [96]. Therefore, it is possible that antioxidants are depleted in the early period after ischemia as a consequence of an excessive production of ROS and increased oxidative stress. Bayir and Kiyici [97] reported that SOD activity levels in the blood were lower in patients with a severe head injury compared to patients with a moderate head injury. Furthermore, patients with medium and large infarcts had lower SOD activity compared to those with less extensive strokes, which may also reflect the increased amount of ROS released from a severe ischemic injury [95]. Interestingly, SOD levels in the CSF or serum of the ischemic cerebrovascular patients increased after two days, reaching the highest values after one week in CSF and two weeks in plasma and returned to initial concentrations after three weeks [98]. The cited data show that changes in SOD activity are observed right after brain injury, and depending on the type of disease, could only be noticeable for a short period of time. In the current study, evaluation of SOD activity in CSF and plasma after BM-MSC therapy was performed two months after transplantation, so it may be supposed that assessment of actual effects of BM-MSC treatment on SOD antioxidant activity was not possible.

The results of the current study revealed increased CAT activity in CSF after the first and second BM-MSC transplantation compared to the control value. Surprisingly, GPx activity, known as a crucial antioxidant enzyme, oscillated at the limit of detection and did not allow reliable analysis to be performed or conclusions to be drawn. The observed changes may imply that CAT was the key enzyme responsible for catalyzing the decomposition of H_2_O_2_ in CSF of patients in MCS. These results are consistent with studies showing that CAT is more efficient in removing higher intracellular H_2_O_2_ concentrations, whereas at low concentrations this radical is disposed mainly by GPx [99,100]. Moreover, conversion of H_2_O_2_ into harmless forms by GPx occurs with the concomitant oxidation of reduced glutathione (GSH) into the oxidized form (GSSG), and glutathione reductase recycles GSSG to GSH. Thus, it is supposed that the main limiting factor for GPx in scavenging higher amounts of H_2_O_2_ is the rate of recycling of GSH/thioredoxin, which is necessary to maintain the catalytic cycle [101,102]. On the other hand, the MSC-treated group of mice with induced oxidative stress accompanying colitis exhibited greater GSH levels than the untreated group and these were similar to that observed in the control group [103]. However, this variance of results in comparison to our data may be caused by different disorders, as well as the different therapy recipients.

It was documented that CAT may be an alternate source for oxygen upon H_2_O_2_ degradation to rescue neurons from hypoxic conditions and revealed the highest turnover rates among known enzymes [104]. Moreover, CAT encapsulated with PLGA (poly (lactic co-glycolic acid)) nanoparticles and added to the primary human neuron cultures protected these cells from H_2_O_2_-mediated cytotoxicity by reducing cellular protein oxidation, DNA damage, loss of neuronal cytoskeleton structure, and plasma membrane integrity [104]. The increased CAT level without significant changes in SOD activity observed in this study may be explained by the fact that enhanced CAT activity reflects elevated levels of H_2_O_2_ produced not only by SOD but also by other cellular mechanisms [105]. Furthermore, the efficacy of SOD in reducing oxidative stress depends predominantly on the elimination of H_2_O_2_ by CAT and GPx [86], so we suggest that in case of BM-MSC therapy, CAT is crucial in oxidative stress regulation. On the other hand, it was documented that an excess of ROS negatively affected the neuroprotective potential of mesenchymal stem cell treatment. The adipose tissue-derived MSC (Ad-MSCs) treatment was less effective alone than in the case of the administration of Ad-MSCs with CuZn-SOD in a rabbit model of spinal cord ischemia [106]. Moreover, oxidative stress-induced impairments in cellular molecules promote stem cells aging [47] and reduce MSC survival [107]; however, the animal experimental model cannot be fully objective in relation to human therapy. Taking the above into consideration, the high ROS level accompanying coma and further states as MCS may also influence MSC properties.

The qualification process of patients presenting disorders of consciousness (DOC) is still under development, and therefore, a number of diagnostic methods analyzing pathomechanism, clinical evaluation, and outcome predictions is required for accurate diagnosis [108]. Despite the approved classification of DOC, the latest studies indicate the necessity for inclusion of a new category of patients with cognitive and motor dissociation (CMD) [109,110]. In this study, we analyzed oxidative stress markers in plasma and CSF of seven patients with MCS caused by head injury and three patients with other MCS etiology. Regardless of differentiated MCS pathomechanism, we gained very homogenous results, without significant standard deviations. The influence of BM-MSC therapy of MCS patients on oxidative stress markers irrespective of the pathomechanism of the disorder seems possible. However, the examined group may not be large enough to evaluate whether the results of the therapy are dependent on the pathomechanism of MCS. Due to the heterogenous etiology of brain lesions, supplementing the manuscript with MRI analysis, performed before and after treatment, would be helpful in precise assessment of the therapy results. Integration of well-known and newly described tools, such as Coma Recovery Scale-Revised (CRS-R), the Motor Behavior Tool (TBM), neuroimaging, and electrophysiological technologies, seems to be essential for the future of effective therapeutic approaches.

## 5. Conclusions

Summarizing, the presence of all examining antioxidants in CSF and plasma of patients undergoing therapy was confirmed, except GPx, both before and after BM-MSC transplantation. A greater content of AA and CAT activity was detected in CSF in comparison to plasma, suggesting the major role of these factors in maintaining the proper level of ROS in the CNS of MCS patients. Moreover, higher total antioxidant ability and lower AA concentration detected after BM-MSC transplantation than before therapy both in CSF and plasma, indicate that BM-MSC administration increased the capacity of CNS cells to ROS utilization and that the important tool of this action is intensively consumed AA. Furthermore, change in CSF:plasma ratio of AA concentration after BM-MSC transplantation indicates that during therapy AA sources were completed by the transport of AA from plasma to CSF. Moreover, CAT activity was changed after BM-MSC transplantation, which suggests that BM-MSC therapy for MCS patients also influenced the enzymatic antioxidant mechanism. The above study indicates the influence of BM-MSC therapy on the oxidative stress intensity in patients in MCS regardless of pathomechanism and clinical evaluation. These results confirm the hypothesis that BM-MSC transplantation is a promising therapeutic tool for neurodegenerative disorders. However, the results of ongoing clinical evaluations are needed to assess the effectiveness of BM-MSC application on the course of recovery in MCS patients.

## Figures and Tables

**Table 1:** Clinical and demographic characteristic of nine patients in minimally conscious state (MCS) in treatment with bone marrow-derived mesenchymal stem cells. The table contains data on age, sex, mechanism of injury, and length of time in the state of minimal consciousness from the time of injury to the start of experimental therapy.

	Patient 1	Patient 2	Patient 3	Patient 4	Patient 5	Patient 6	Patient 7	Patient 8	Patient 9
Age (years)	22	19	45	19	30	23	36	35	38
Gender (M/F)	M	M	M	M	F	F	F	F	M
Mechanism of MCS induction	Head injury after traffic accident	Head injury after traffic accident	Head injury after traffic accident	Head injury after traffic accident	Head injury after traffic accident	Listeriosis encephalitis with damage to the thalamus and basal ganglia	Ischemia with cardiac arrest	Hypoglycemia after poisoning	Head injury after trafficaccident
Time in MCS before the clinical trial (months)	3	8	7	6	12	10	14	6	6

**Table 2:** Total antioxidant capacity (A), ascorbic concentration (B), total ascorbate concentration (C), super oxide dismutase (D), and catalase (E) activity in CSF and plasma in patients in MCS before and after BM-MSC therapy. Small letters a and b indicate statistical differences in the measured parameters between CSF and plasma within the control, first, and second transplantation of BM-MSC. The statistical analysis was determined by Student’s t-test (*p* < 0.0001).

		Before Treatment		I Application of MSC		II Application of MSC	
		CSF	plasma	CSF	plasma	CSF	plasma
A	Total antioxidant capacity (nM)	0.05 ± 0.015 **^a^**	0.39 ± 0.04 **^b^**	0.08 ± 0.014 **^a^**	0.34 ± 0.04 **^b^**	0.08 ± 0.012 **^a^**	0.39 ± 0.04 **^b^**
							
B	Ascorbic acid concentration (ng/μL)	27.25 ± 1.31 **^a^**	9.96 ± 1.44 **^b^**	22.73 ± 1.42 **^a^**	6.39 ± 1.32 **^b^**	28.13 ± 1.22 **^a^**	7.36 ± 1.56 **^b^**
							
C	Total ascorbate concentration (nM/μL)	0.20 ± 0.009 **^a^**	0.39 ± 0.02 **^b^**	0.17 ± 0.008 **^a^**	0.32 ± 0.02 **^b^**	0.19 ± 0.01 **^a^**	0.32 ± 0.02 **^b^**
							
D	Superoxide dismutase (SOD) activity (%)	40.82 ± 2.05 **^a^**	65.66 ± 2.27 **^b^**	37.42 ± 1.97 **^a^**	66.35 ±2.27 **^b^**	36.87 ± 2.11 **^a^**	60.73 ± 2.41 **^b^**
							
E	Catalase (CAT) activity (μmol/min/mL)	11.29 ± 0.60 **^a^**	0.04 ± 0.008 **^b^**	13.20 ± 0.51 **^a^**	0.06 ± 0.008 **^b^**	12.38 ± 0.59 **^a^**	0.03 ± 0.009 **^b^**

## References

[1] Wijdicks E.F.M. (2010). The bare essentials: Coma. Pract. Neurol..

[2] Traub S.J., Wijdicks E.F. (2016). Initial Diagnosis and Management of Coma. Emerg. Med. Clin. North Am..

[3] Ludwig L., McWhirter L., Williams S., Derry C., Stone J. (2016). Functional coma. Handb. Clin. Neurol..

[4] Giacino J.T., Ashwal S., Childs N., Cranford R., Jennett B., Katz D.I., Kelly J.P., Rosenberg J.H., Whyte J., Zafonte R.D. (2002). The minimally conscious state: Definition and diagnostic criteria. Neurology.

[5] Bhat A.H., Dar K.B., Anees S., Zargar M.A., Masood A., Sofi M.A., Ganie S.A. (2015). Oxidative stress, mitochondrial dysfunction and neurodegenerative diseases; a mechanistic insight. Biomed. Pharmacother..

[6] Islam M.T. (2016). Oxidative stress and mitochondrial dysfunction-linked neurodegenerative disorders. Neurol. Res..

[7] Salim S. (2017). Oxidative Stress and the Central Nervous System. J. Pharmacol. Exp. Ther..

[8] Angelova P.R., Abramov A.Y. (2018). Role of mitochondrial ROS in the brain: From physiology to neurodegeneration. FEBS Lett..

[9] Nissanka N., Moraes C.T. (2017). Mitochondrial DNA damage and reactive oxygen species in neurodegenerative disease. FEBS Lett..

[10] Zhuo M., Small S.A., Kandel E.R., Hawkins R.D. (1993). Nitric oxide and carbon monoxide produce activity-dependent long-term synaptic enhancement in hippocampus. Science.

[11] Massaad C.A., Klann E. (2011). Reactive Oxygen Species in the Regulation of Synaptic Plasticity and Memory. Antioxid. Redox. Signal..

[12] O’Dell T.J., Hawkins R.D., Kandel E.R., Arancio O. (1991). Tests of the roles of two diffusible substances in long-term potentiation: Evidence for nitric oxide as a possible early retrograde messenger. Proc. Natl. Acad. Sci. USA.

[13] Verma A., Hirsch D.J., Glatt C.E., Ronnett G.V., Snyder S.H. (1993). Carbon monoxide: A putative neural messenger. Science.

[14] Knapp L.T., Klann E. (2002). Role of reactive oxygen species in hippocampal long-term potentiation: Contributory or inhibitory?. J. Neurosci. Res..

[15] Kohen R., Beit-Yannai E., Berry E.M., Tirosh O. (1999). Overall low molecular weight antioxidant activity of biological fluids and tissues by cyclic voltammetry. Meth. Enzymol..

[16] Kohen R., Vellaichamy E., Hrbac J., Gati I., Tirosh O. (2000). Quantification of the overall reactive oxygen species scavenging capacity of biological fluids and tissues. Free Radic. Biol. Med..

[17] Nualart F., Mack L., García A., Cisternas P., Bongarzone E.R., Heitzer M., Jara N., Martínez F., Ferrada L., Espinoza F. (2014). Vitamin C Transporters, Recycling and the Bystander Effect in the Nervous System: SVCT2 versus Gluts. J. Stem Cell Res. Ther..

[18] Chance B., Schoener B., Oshino R., Itshak F., Nakase Y. (1979). Oxidation-reduction ratio studies of mitochondria in freeze-trapped samples. NADH and flavoprotein fluorescence signals. J. Biol. Chem..

[19] Griendling K.K., Sorescu D., Lassègue B., Ushio-Fukai M. (2000). Modulation of protein kinase activity and gene expression by reactive oxygen species and their role in vascular physiology and pathophysiology. Arterioscler. Thromb. Vasc. Biol..

[20] Saso L., Firuzi O. (2014). Pharmacological applications of antioxidants: Lights and shadows. Curr. Drug Targets.

[21] Strauss D.J., Ashal S., Day S.M., Schavelle M.R. (2000). Life expectancy of children in vegetative and minimally conscious states. Pediatr. Neurol..

[22] Laureys S., Owen A.M., Schiff N.D. (2007). Behavioural improvements with thalamic stimulation after severe traumatic brain injury. Nature.

[23] Angelakis E., Liouta E., Andreadis N., Korfias S., Ktonas P., Stranjalis G., Sakas D.E. (2014). Transcranial direct current stimulation (tDCS) effects in disorders of consciousness. Arch. Phys. Med. Rehabil..

[24] Thibaut A., Bruno M.A., Ledoux D., Demertzi A., Laureys S. (2014). tDCS in patients with disorders of consciousness: Sham-controlled randomized double-blind study. Neurology.

[25] Le Blanc K., Ringden O. (2005). Immunobiology of human mesenchymal stem cells and future use in hematopoietic stem cell transplantation. Am. Soc. Blood Marrow Trans..

[26] Yokokawa K., Iwahara N., Hisahara S., Emoto M.C., Saito T., Suzuki H., Manabe T., Matsumura A., Matsushita T., Suzuki S. (2019). Transplantation of Mesenchymal Stem Cells Improves Amyloid-β Pathology by Modifying Microglial Function and Suppressing Oxidative Stress. J. Alzheimers Dis..

[27] Kurozumi K., Nakamura K., Tamiya T., Kawano Y., Ishii K., Kobune M., Hirai S., Uchida H., Sasaki K., Ito Y. (2005). Mesenchymal stem cells that produce neurotrophic factors reduce ischemic damage in the rat middle cerebral artery occlusion model. Mol. Ther..

[28] Sharma S., Yang B., Strong R., Xi X., Brenneman M., Grotta J.C., Aronowski J., Savitz S.I. (2010). Bone marrow mononuclear cells protect neurons and modulate microglia in cell culture models of ischemic stroke. J. Neurosci. Res..

[29] Savitz S.I., Misra V., Kasam M., Juneja H., Cox C.S., Alderman S. (2011). Intravenous autologous bone marrow mononuclear cells for ischemic stroke. Ann. Neurol..

[30] Galgano M., Toshkezi G., Qiu X., Russell T., Chin L., Zhao L.R. (2017). Traumatic Brain Injury: Current Treatment Strategies and Future Endeavors. Cell Transplant..

[31] Wilkins A., Kemp K., Ginty M., Hares K., Mallam E., Scolding N. (2009). Human bone marrow-derived mesenchymal stem cells secrete brain-derived neurotrophic factor which promotes neuronal survival in vitro. Stem Cell Res..

[32] Bao X., Feng M., Wei J., Han Q., Zhao H., Li G., Zhu Z., Xing H., An Y., Qin C. (2011). Transplantation of Flk-1+ human bone marrow-derived mesenchymal stem cells promote angiogenesis and neurogenesis after cerebral ischemia in rats. Eur. J. Neurosci..

[33] Bedi S.S., Walker P.A., Shah S.K., Jimenez F., Thomas C.P., Smith P., Hetz R.A., Xue H., Pati S., Dash P.K. (2013). Autologous bone marrow mononuclear cells therapy attenuates activated microglial/macrophageresponse and improves spatial learning after traumatic brain injury. J. Trauma Acute Care Surg..

[34] Urao N., Ushio-Fukai M. (2013). Redox regulation of stem/progenitor cells and bone marrow niche. Free Rad. Biol. Med..

[35] Ryu J.M., Lee H.J., Jung Y.H., Lee K.H., Kim D.I., Kim J.Y., Ko S.H., Choi G.E., Chai I.I., Song E.J. (2015). Regulation of stem cell fate by ROS-mediated alteration of metabolism. Int. J. Stem Cells.

[36] Brenneman M., Sharma S., Harting M., Strong R., Cox C.S., Aronowski J., Grotta J.C., Savitz S.I. (2010). Autologous bone marrow mononuclear cells enhance recovery after acute ischemic stroke in young and middle-aged rats. J. Cereb. Blood Flow Metab..

[37] Wakabayashi K., Nagai A., Sheikh A.M., Shiota Y., Narantuya D., Watanabe T., Masuda J., Kobayashi S., Kim S.U., Yamaguchi S. (2010). Transplantation of human mesenchymal stem cells promotes functional improvement and increased expression of neurotrophic factors in a rat focal cerebral ischemia model. J. Neurosci. Res..

[38] Chen M.F., Lin C.T., Chen W.C., Yang C.T., Chen C.C., Liao S.K., Liu J.M., Lu C.H., Lee K.D. (2006). The sensitivity of human mesenchymal stem cells to ionizing radiation. Int. J. Radiat. Oncol. Biol. Phys..

[39] Valle-Prieto A., Conget P.A. (2010). Human Mesenchymal Stem Cells Efficiently Manage Oxidative Stress. Stem Cells Dev..

[40] Grisendi G., Annerén C., Cafarelli L., Sternieri R., Veronesi E., Cervo G.L., Luminari S., Maur M., Frassoldati A., Palazzi G. (2010). GMP-manufactured density gradient media for optimized mesenchymal stromal/stem cell isolation and expansion. Cytotherapy.

[41] Dominici M., Le Blanc K., Mueller I., Slaper-Cortenbach I., Marini F. (2006). Minimal criteria for defining multipotent mesenchymal stromal cells. Cytotherapy.

[42] Maehly A.C., Chance B. (1954). The assay of catalases and peroxidases. Methods Biochem. Anal..

[43] Shannon L.M., Kay E., Lew J.Y. (1966). Peroxidase isoenzymes from horseradish roots. I. Isolation and physical properties. J. Biol. Chem..

[44] Bayır H., Kochanek P.M., Kagan V.E. (2006). Oxidative Stress in Immature Brain after Traumatic Brain Injury. Dev. Neurosci..

[45] Lagowska-Lenard M., Bielewicz J., Raszewski G., Stelmasiak Z., Bartosik-Psujek H. (2008). Oxidative stress in cerebral stroke. Pol. Merk. Lek..

[46] Schieber M., Chandel N.S. (2014). ROS function in redox signaling and oxidative stress. Curr. Biol..

[47] Chen F., Liu Y., Wong N.K., Xiao J., So K.F. (2017). Oxidative Stress in Stem Cell Aging. Cell Transplant..

[48] Carvalho A.N., Firuzi O., Gama M.J., Horssen J.V., Saso L. (2017). Oxidative Stress and Antioxidants in Neurological Diseases: Is There Still Hope?. Curr. Drug Targets.

[49] Rebec G.V., Pierce R.C. (1994). A vitamin as neuromodulator: Ascorbate release into the extracellular fluid of the brain regulates dopaminergic and glutamatergic transmission. Prog. Neurobiol..

[50] Karanth S., Yu W.H., Walczewska A., Mastronardi C., McCann S.M. (2000). Ascorbic acid acts as an inhibitory transmitter in the hypothalamus to inhibit stimulated luteinizing hormone-releasing hormone release by scavenging nitric oxide. Proc. Natl. Acad. Sci. USA.

[51] Qiu S., Li L., Weeber E.J., May J.M. (2007). Ascorbate transport by primary cultured neurons and its role in neuronal function and protection against excitotoxicity. J. Neurosci. Res..

[52] Pastor P., Cisternas P., Salazar K., Silva-Alvarez C., Oyarce K., Jara N., Espinoza F., Martínez A.D., Nualart F. (2013). SVCT2 vitamin C transporter expression in progenitor cells of the postnatal neurogenic niche. Front Cell Neurosci..

[53] Farbstein D., Kozak -Blickstein A., Levy A.P. (2010). Antioxidant vitamins and their use in preventing cardiovascular disease. Molecules.

[54] Spector R., Lorenzo A.V. (1973). Ascorbic acid homeostasis in the central nervous system. Am. J. Physiol..

[55] Englard S., Seifter S. (1986). The biochemical functions of ascorbic acid. Annu. Rev. Nutr..

[56] Rice M.E. (2000). Ascorbate regulation and its neuroprotective role in the brain. Trends Neurosci..

[57] DeMenezes C.C., Dorneles A.G., Sperotto R.L., Duarte M.M., Schetinger M.R., Loro V.L. (2009). Oxidative stress in cerebrospinal fluid of patients with aseptic and bacterial meningitis. Neurochem. Res..

[58] Bowman G.L., Dodge H., Frei B., Calabrese C., Oken B.S., Kaye J.A., Quinn J.F. (2009). Ascorbic acid and rates of cognitive decline in Alzheimer’s disease. J. Alzheimers Dis..

[59] Arlt S., Müller-Thomsen T., Beisiegel U., Kontush A. (2012). Effect of one-year vitamin C- and E-supplementation on cerebrospinal fluid oxidation parametersand clinical course in Alzheimer’s disease. Neurochem. Res..

[60] Prasad R., Mishra O.P., Mishra S.P., Upadhyay R.S., Singh T.B. (2012). Oxidative stress in children with neurocysticercosis. Pediatr. Infect. Dis. J..

[61] Arlt S., Kontush A., Zerr I., Buhmann C., Jacobi C., Schröter A., Poser S., Beisiegel U. (2002). Increased lipid peroxidation in cerebrospinal fluid and plasma from patients with Creutzfeldt-Jakob disease. Neurobiol. Dis..

[62] Voigt K., Kontush A., Stuerenburg H.J., Muench-Harrach D., Hansen H.C., Kunze K. (2002). Decreased plasma and cerebrospinal fluid ascorbate levels in patients with septic encephalopathy. Free Radic Res..

[63] Paraskevas G.P., Kapaki E., Libitaki G., Zournas C., Segditsa I., Papageorgiou C. (1997). Ascorbate in healthy subjects, amyotrophic lateral sclerosis and Alzheimer’s disease. Acta Neurol. Scand..

[64] Buhmann C., Arlt S., Kontush A., Möller-Bertram T., Sperber S., Oechsner M., Stuerenburg H.J., Beisiegel U. (2004). Plasma and CSF markers of oxidative stress are increased in Parkinson’s disease and influenced by antiparkinsonian medication. Neurobiol. Dis..

[65] Reiber H., Ruff M., Uhr M. (1993). Ascorbate concentration in human cerebrospinal fluid (CSF) and serum. Intrathecal accumulation and CSF flow rate. Clin. Chim. Acta.

[66] Tallaksen C.M., Bøhmer T., Bell H. (1992). Concentrations of the water-soluble vitamins thiamin, ascorbic acid, and folic acid in serum and cerebrospinal fluid of healthy individuals. Am. J. Clin. Nutr..

[67] Brau R.H., García-Castiñeiras S., Rifkinson N. (1984). Cerebrospinal fluid ascorbic acid levels in neurological disorders. Neurosurgery.

[68] Polidori M.C., Mecocci P., Frei B. (2001). Plasma vitamin C levels are decreased and correlated with brain damage in patients with intracranial hemorrhage or head trauma. Stroke.

[69] Rice M.E., Russo-Menna I. (1998). Differential compartmentalization of brain ascorbate and glutathione between neurons and glia. Neuroscience.

[70] Nayak C., Nayak D., Raja A., Rao A. (2006). Time-level relationship between indicators of oxidative stress and Glasgow Coma Scale scores of severe head injury patients. Clin. Chem. Lab. Med..

[71] Alho H., Leinonen J.S., Erhola M., Lönnrot K., Aejmelaeus R. (1998). Assay of antioxidant capacity of human plasma and CSF in aging and disease. Restor. Neurol. Neurosci..

[72] Liu J.T., Tan W.C., Liao W.J. (2008). Effects of electrical cervical spinal cord stimulation on cerebral blood perfusion, cerebrospinal fluid catecholamine levels, and oxidative stress in comatose patients. Acta Neurochir. Suppl..

[73] Spector R., Spector A.Z., Snodgrass S.R. (1977). Model for transport in the central nervous system. Am. J. Physiol..

[74] Spector R., Johanson C.E. (2014). The nexus of vitamin homeostasis and DNA synthesis and modification in mammalian brain. Mol. Brain.

[75] Angelow S., Haselbach M., Galla H.J. (2003). Functional characterisation of the active ascorbic acid transport into cerebrospinal fluid using primary cultured choroid plexus cells. Brain Res..

[76] Nualart F., Salazar K., Oyarce K., Cisternas P., Jara N., Silva-Álvarez C., Pastor P., Martínez F., García A., García-Robles Mde L. (2012). Typical and atypical stem cells in the brain, vitamin C effect and neuropathology. Biol. Res..

[77] May J.M. (2012). Vitamin C transport and its role in the central nervous system. Subcell. Bochem..

[78] Vera J.C., Rivas C.I., Fischbarg J., Golde D.W. (1993). Mammalian facilitative hexose transporters mediate the transport of dehydroascorbic acid. Nature.

[79] Rumsey S.C., Kwon O., Xu G.W., Burant C.F., Simpson I., Levine M. (1997). Glucose transporter isoforms GLUT1 and GLUT3 transport dehydroascorbic acid. J. Biol. Chem..

[80] Cisternas P., Silva-Alvarez C., Martinez F., Fernandez E., Ferrada L., Oyarce K., Salazar K., Bolaños J.P., Nualart F. (2014). The oxidized form of vitamin C, dehydroascorbic acid, regulates neuronal energy metabolism. J. Neurochem..

[81] Astuya A., Caprile T., Castro M., Salazar K., de Garcia M.L., Reinicke K., Rodríguez F., Vera J.C., Millán C., Ulloa V. (2005). Vitamin C uptake and recycling among normal and tumor cells from the central nervous system. J. Neurosci. Res..

[82] Castro M.A., Pozo M., Cortes C., de Garcia M.L., Concha I.I., Nualart F. (2007). Intracellular ascorbic acid inhibits transport of glucose by neurons, but not by astrocytes. J. Neurochem..

[83] Arlt S., Müller-Thomsen T., Beisiegel U. (2002). Use of Vitamin C and E in the Treatment of Alzheimer’s Disease. Drug Dev. Res..

[84] Schippling S., Kontush A., Arlt S., Buhmann C., Stürenburg H.J., Mann U., Müller-Thomsen T., Beisiegelm U. (2000). Increased lipoprotein oxidation in Alzheimer’s disease. Free Radic Biol. Med..

[85] Aygul R., Kotan D., Demirbas F., Ulvi H., Deniz O. (2006). Plasma oxidants and antioxidants in acute ischemic stroke. J. Int. Med. Res..

[86] Warner D.S., Sheng H., Batinic-Haberle I. (2004). Oxidants, antioxidants and the ischemic brain. J. Exp. Biol..

[87] Jacobsson J., Jonsson P.A., Andersen P.M., Forsgren L., Marklund S.L. (2001). Superoxide dismutase in CSF from amyotrophic lateral sclerosis patients with and without CuZn-superoxide dismutase mutations. Brain.

[88] Younus H. (2018). Therapeutic potentials of superoxide dismutase. Int. J. Health Sci. (Qassim).

[89] Strand T., Marklund S.L. (1992). Release of Superoxide Dismutase into Cerebrospinal Fluid as a Marker of Brain Lesion in Acute Cerebral Infarction. Stroke.

[90] Yoshida E., Mokuno K., Aoki S., Takahashi A., Riku S., Murayama T., Yanagi T., Kato K. (1994). Cerebrospinal fluid levels of superoxide dismutases in neurological diseases detected by sensitive enzyme immunoassays. J. Neurol. Sci..

[91] Kemp K., Hares K., Mallam E., Heesom K.J., Scolding N., Wilkins A. (2010). Mesenchymal stem cell-secreted superoxide dismutase promotes cerebellar neuronal survival. J. Neurochem..

[92] Whone A.L., Kemp K., Suna M., Wilkins A., Scolding N.J. (2012). Human bone marrow mesenchymal stem cells protect catecholaminergic and serotonergic neuronal perikarya and transporter function from oxidative stress by the secretion of glial-derived neurotrophic factor. Brain Res..

[93] Alhazzani A., Rajagopalan P., Albarqi Z., Devaraj A., Mohamed M.H., Al-Hakami A., Chandramoorthy H.C. (2018). Mesenchymal Stem Cells (MSCs) Coculture Protects [Ca2+]i Orchestrated Oxidant Mediated Damage in Differentiated Neurons In Vitro. Cells.

[94] Sapoynikova N., Asatiani N., Kartvelishvili T., Kalandadze I., Tsiskaridze A., El-Missiry M.A. (2012). Plasma Antioxidant Activity as a Marker for a Favourable Outcome in Acute Ischemic Stroke. Antioxidant Enzyme.

[95] Spranger M., Krempien S., Schwab S., Donneberg S., Werner H. (1997). Superoxide Dismutase Activity in Serum of Patients with Acute Cerebral Ischemic Injury. Correlation with Clinical Course and Infarct Size. Stroke.

[96] Kaynar M.Y., Taner T., Rahsan K., Pinar A., Gumustas K. (2005). Cerebrospinal fluid superoxide dismutase and serum malondialdehyde levels in patients with aneurysmal subarachnoid hemorrhage: Preliminary results. Neurol. Res..

[97] Bayır A., Kıyıcı A. (2011). The Relation between Glasgow Coma Score and Blood Superoxide Dismutase Activity in Patients with Traumatic Brain Injury. J. Head Trauma Rehabil..

[98] Gruener N., Gross B., Gozlan O., Barak M. (1994). Increase in superoxide dismutase after cerebrovascular accident. Life Sci..

[99] Baud O., Greene A.E., Li J., Wang H., Volpe J.J. (2004). Rosenberg PA. Glutathione peroxidase-catalase cooperativity is required for resistance to hydrogen peroxide by mature rat oligodendrocytes. J. Neurosci..

[100] Kodydková J., Vávrová L., Kocík M., Žák A. (2014). Human catalase, its polymorphisms, regulation and changes of its activity in different diseases. Folia. Biol. (Praha).

[101] Cornelius C., Crupi R., Calabrese V., Graziano A., Milone P., Pennisi G., Radak Z., Calabrese E.J., Cuzzocrea S. (2013). Traumatic brain injury: Oxidative stress and neuroprotection. Antioxid. Redox Signal..

[102] Gebicka L., Krych-Madej J. (2019). The role of catalases in the prevention/promotion of oxidative stress. J. Inorg. Biochem..

[103] Da Costa Gonçalves F., Grings M., Nunes N.S., Pinto F.O., Garcez T.N., Visioli F., Leipnitz G., Paz A.H. (2017). Antioxidant properties of mesenchymal stem cells against oxidative stress in a murine model of colitis. Biotechnol. Lett..

[104] Singhal A., Morris V.B., Labhasetwar V., Ghorpade A. (2013). Nanoparticle-mediated catalase delivery protects human neurons from oxidative stress. Cell Death Dis..

[105] Lanza C., Morando S., Voci A., Canesi L., Principato M.C., Serpero L.D., Mancardi G., Uccelli A., Vergani L. (2009). Neuroprotective mesenchymal stem cells are endowed with a potent antioxidant effect in vivo. J. Neurochem..

[106] Yoo D.Y., Kim D.W., Chung J.Y., Jung H.Y., Kim J.W., Yoon Y.S., Hwang I.K., Choi J.H., Choi G.M., Choi S.Y. (2016). Cu, Zn-Superoxide Dismutase Increases the Therapeutic Potential of Adipose-derived Mesenchymal Stem Cells by Maintaining Antioxidant Enzyme Levels. Neurochem. Res..

[107] Xu J., Huang Z., Lin L., Fu M., Gao Y., Shen Y., Zou Y., Sun A., Qian J., Ge J. (2014). miR-210 over-expression enhances mesenchymal stem cell survival in an oxidative stress environment through antioxidation and c-Met pathway activation. Sci. China Life Sci..

[108] Pincherle J.J., Chatelle C., Pignat J.M., Du Pasquier R., Ryvlin P., Oddo M., Diserens K. (2019). Motor behavior unmasks residual cognition in disorders of consciousness. Ann. Neurol..

[109] Schiff N.D. (2015). Cognitive Motor Dissociation Following Severe Brain Injuries. JAMA Neurol..

[110] Pignat J.M., Mauron E., Jöhr J., Gilart de Keranflec’h C., Van De Ville D., Preti M.G., Meskaldji D.E., Hömberg V., Laureys S., Draganski B. (2016). Outcome Prediction of Consciousness Disorders in the Acute Stage Based on a Complementary Motor Behavioural Tool. PLoS ONE.

